# Supplementary material for: Engineering circular guide RNA and CRISPR-Cas13d-encoding mRNA for the RNA editing of Adar1 in triple-negative breast cancer immunotherapy
Source: bioRxiv. 2025 Jul 22:2025.07.22.666181. Preprint. [Version 1] doi: 10.1101/2025.07.22.666181 (PMC12330674; doi:10.1101/2025.07.22.666181)
Supplement: Supplement 1 [file media-1.pdf]

Supplementary Information

Engineering circular guide RNA and CRISPR-Cas13d-encoding mRNA for the RNA editing of *Adar1* in triple-negative breast cancer immunotherapy

Shurong Zhou[1], Suling Yang[1], Jie Xu[2], Guizhi Zhu[1,3] *

[1] Department of Pharmaceutical Sciences, College of Pharmacy, University of Michigan, Ann Arbor, MI 48109, USA

[2] Center for Advanced Models for Translational Sciences and Therapeutics, University of Michigan Medical Center, Ann Arbor, Michigan, 48109, USA

[3] Bioinnovations in Brain Cancer; Biointerfaces Institute; Rogel Cancer Center; Center for RNA Biomedicine. University of Michigan, Ann Arbor, MI 48109, USA

* Corresponding authors (G.Z.: guizhiz@umich.edu)

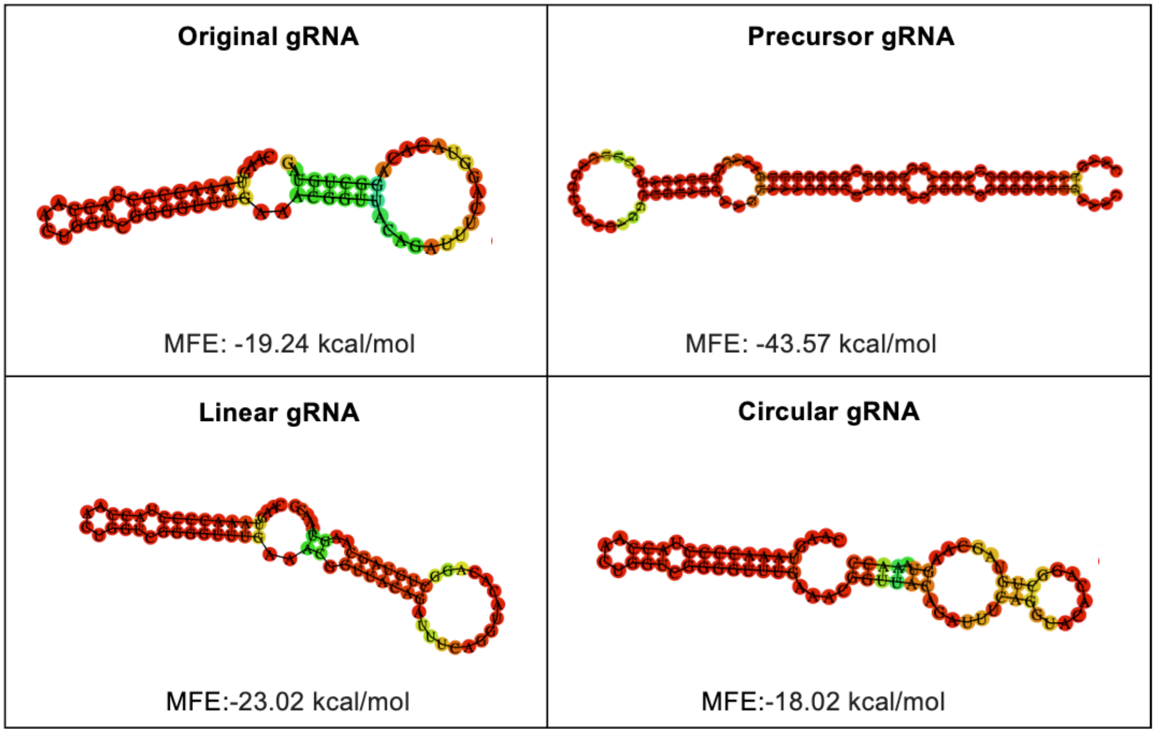

Figure S1. Predicted secondary structures of RNA sequences using RNAfold (http://rna.tbi.univie.ac.at/cgi-bin/RNAWebSuite/RNAfold.cgi).

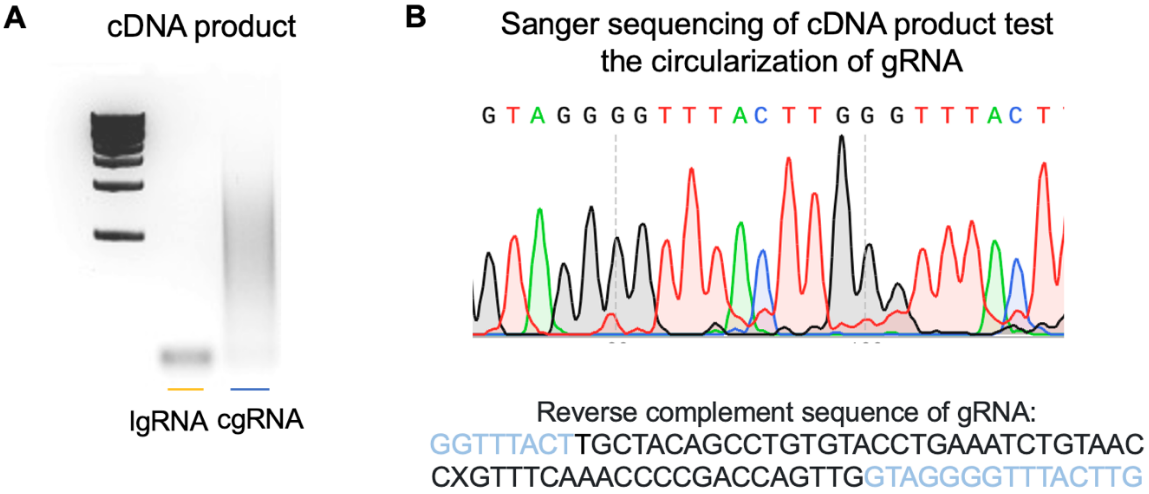

Figure S2. Sanger sequencing of the cDNA of cgRNA junction site validates cgRNA synthesis. (A) An image of agarose gel electrophoresis for reverse-transcribed cDNA of cgRNA junction site. (B) Sanger sequencing results of the above reverse-transcribed cDNA.

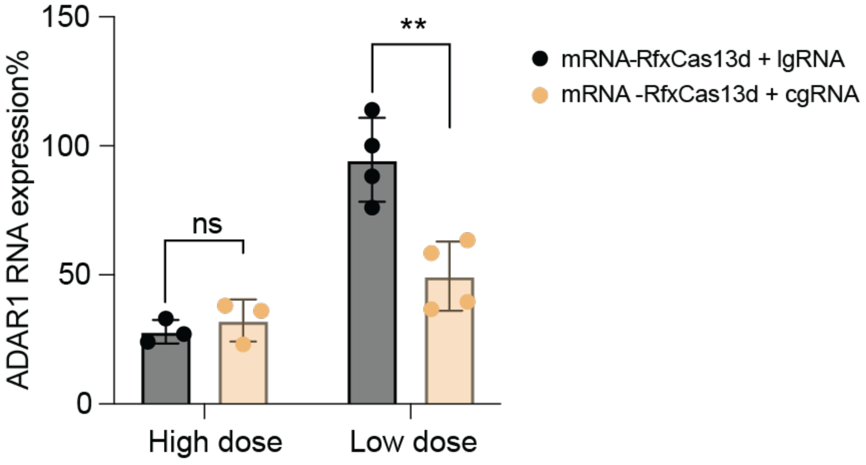

Figure S3. *Adar1* knockdown efficiency using mRNA-RfxCas13d (0.5 μg/mL) together with lgRNA or cgRNA (High dose: 0.5 μg/mL; Low dose: 0.25 μg/mL), respectively, in 4T1 cells at different gRNA concentrations for 24 h. RNA was transfected using Lipofectamine 2000.

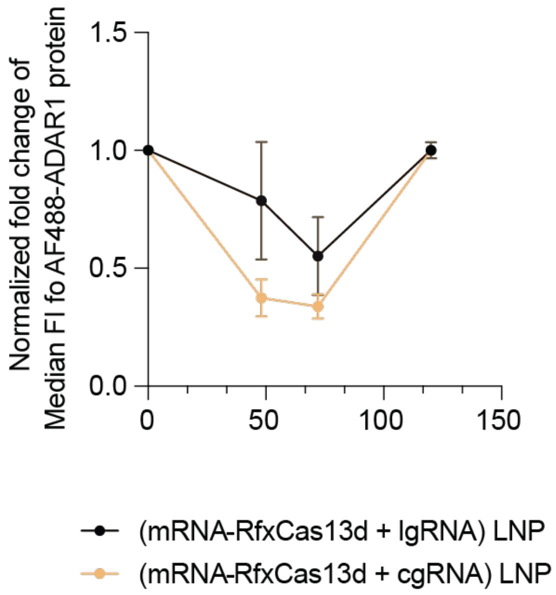

Figure S4. ADAR1 protein expression kinetics in MC38 cells before and after LNP co-transfection of mRNA-RfxCas13d together with lgRNA or cgRNA, respectively.

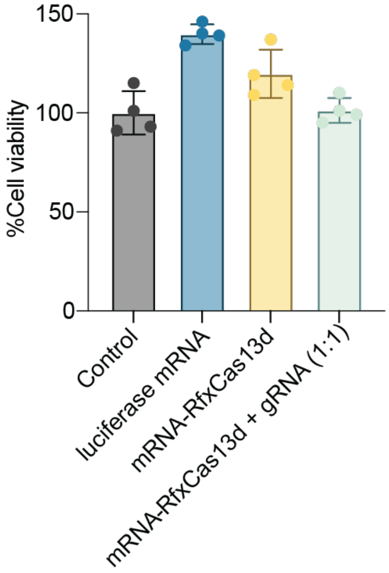

Figure S5. 4T1 cell viability 48 h after mRNA-RfxCas13d (0.5 µg/mL) and gRNA (0.5 µg/mL) co-transfection.

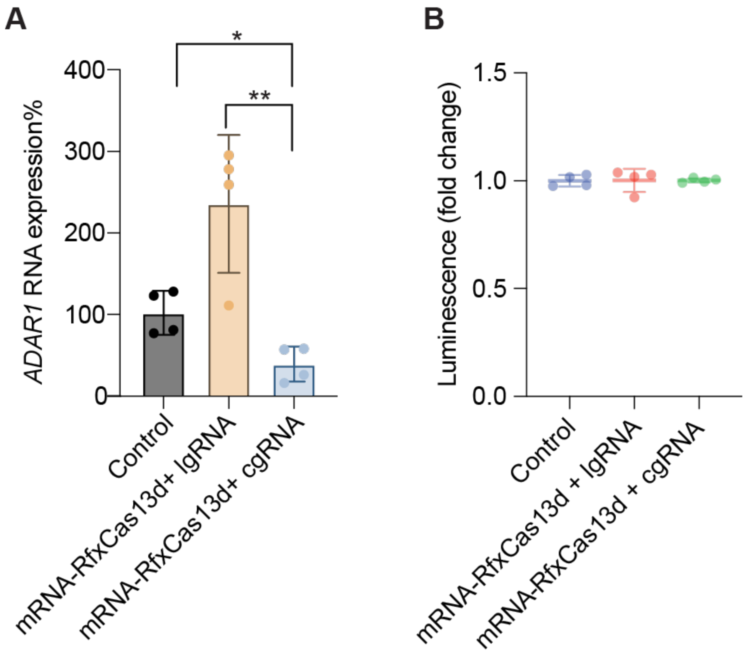

Figure S6. mRNA-RfxCas13d + cgRNA showed efficient *ADAR1*-specific knockdown in human cells with undetectable collateral activities. A) *ADAR1* knockdown efficiency in human MDA-MB 231TNBC cells with a luciferase reporter. B) Evaluation of the collateral activity of the above treatments in luciferase-expressing MDA-MB 231TNBC cells via assessing the activities of the luciferase reporter to generate bioluminescence in the presence of D-luciferin substrate. mRNA-RfxCas13d (0.5 µg/mL) and gRNA (0.5 µg/mL) was co-transfected using lipofectamine 2000 for 24 h.

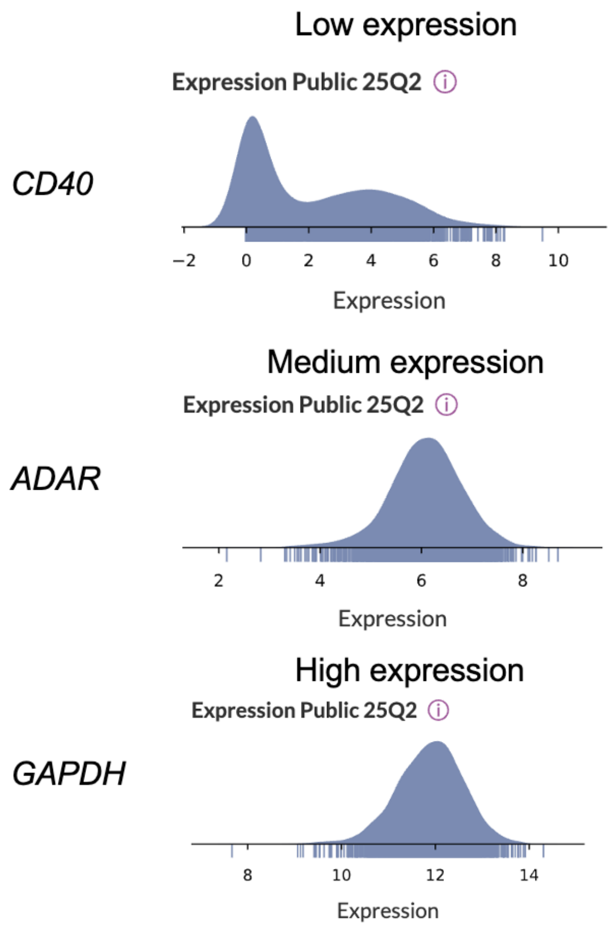

Figure S7. Classification of gene expression level in cancer cells from DepMap Public 25Q2 Dataset.

Table S1. Characterization of RNA-loaded LNPs by dynamic light scattering. PDI: polydispersity index.

LNPs	Diameters/nm	PDI
(mRNA-RfxCas13d + lgRNA) LNPs	104.1± 18	0.14 ± 0.03
(mRNA-RfxCas13d + cgRNA) LNPs	136.7 ± 29	0.18 ± 0.05
